# The impact of COVID‐19 confinement on the eating habits and lifestyle changes: A cross sectional study

**DOI:** 10.1002/fsn3.2179

**Published:** 2021-02-16

**Authors:** Yaseen Galali

**Affiliations:** ^1^ Food Technology Department College of Agricultural Engineering Sciences Salahaddin University‐Erbil Erbil Iraq; ^2^ Department of Nutrition and Dietetics Cihan University‐Erbil Erbil Iraq

**Keywords:** COVID‐19 confinement, eating habit, life style, Mediterranean diet adherence

## Abstract

Novel coronavirus (COVID‐19) emerged in December 2019 in the city of Wuhan, China causing severe respiratory infections and resulting in millions admissions to hospital and over a million deaths worldwide. Authorities imposed protective measures including confinement to flatten curves of casualties resulted in sudden lifestyle and eating habit changes. The objectives of this study were to observe the impact of the COVID‐19 lockdown on eating behavior and lifestyle of the Kurdish population in Iraqi Kurdistan. A structured cross‐sectional study was conducted from 1st to 14th of June when the lockdown finished with three different sections. Section one (6 questions) included socio‐demographic information section two (11 questions) composed of dietary behavior information including a) Mediterranean diet (MD) adherence using 14‐scaled items with a MD screener tool ranging from 0 to 14 b) and sections three (12 questions) composed of lifestyle habit changes such as physical activity, sleeping behavior, and smoking habit. The total number of included respondents was 2,137. The results showed that 12.0% (256) of participants stated that their lifestyle was improved, whereas 50.9% (1,087) declared their lifestyle deteriorated. In particular, the frequency of physical activity was decreased (McNemar value = 60.53, *p* <.001) and sleeping hours were significantly increased during lockdown (McNemar value = 447.93, *p* <.001). Regarding eating habits, it was noticed that 29.3% and 14.3% felt that appetite increased and decreased, respectively. The appetite change during lockdown was significantly associated with age (*p* =.0001), gender (*p* =.001), city (*p* =.007), and BMI (*p* =.001). Similarly, 32.4% reported weight gain. In conclusion, this study is among the earliest studies showing the effect of COVID‐19 on eating behavior and lifestyle changes. COVID‐19 confinement had a significant effect on lifestyle particularly reducing physical activity and changing eating habits.

## Graphical abstract

This article for the first time investigates the influence of COVID‐19 condiment on eating habit and lifestyle in Kurdish population.

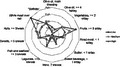

## 1 INTRODUCTION

The novel coronavirus (COVID‐19) emergence in the Chinese city of Wuhan in 2019 spread very rapidly worldwide and caused 1.4 millions deaths in the world (Wang et al., 2020). At the moment, the global cases have passed 80 million with more than 1.76 million deaths. In Iraqi Kurdistan, at the beginning the cases were diagnosed with simple symptoms such as high fever. Later, the severity of the cases was increased and caused 15, 577 infections and deaths reached 597. To decrease the spread of the virus and take physical distancing as recommended, the health authorities introduced lockdown at the beginning of March and sporadically in April 2020 and banned social gatherings and meetings except for the urgent requirements.

There are two major changes that occur when people are quarantined and stayed home and do not practice normal life. The first change includes lifestyle is a reduction in physical activity due to restrictions on travel and sports. Homestay can lead to substantial changes in lifestyle including more sedentary lifestyle, sleeping, and smoking behavior. These changes might adversely affect health. The other change is eating behavior (i.e., stockpiling food and positive energy intake and/or eating disorder; Di Renzo, Gualtieri, Pivari, et al., 2020). During the lockdown, there was limited access to fresh food and a reduction in variety of food groups (Hobbs, 2020). Instead, people might turn to more processed convenient foods products which can be high in energy and low in nutrients. Besides that, the boredom caused by reduction or loss of work (Feather, 1997) and media reports on coronavirus (Gao et al., 2020) makes life stressful. Boredom or stress can lead to emotional, overeating, and high energy food craving (Moynihan et al., 2015; Penaforte et al., 2019) and ultimately adversely affect well‐being.

Therefore, lifestyle and dietary behavior changes were to be expected because of the COVID‐19 lockdown. However, a healthy diet and lifestyle is helpful to support health and well‐being. For this reason, several health organizations including the World Health Organization and European Federation of the Association of Dietitians offered several nutritional and lifestyle recommendations to follow during lockdown (Rodríguez‐Pérez et al., 2020). In particular, the Mediterranean diet (MD) has been proposed as a healthy and immune supportive diet pattern (Muscogiuri et al., 2020).

Therefore, the aim of this study was to assess the influence of COVID‐19 lockdown on the dietary behavior and lifestyle changes in Kurdish populations. For that purpose, a web‐based study was conducted after the lookdown was finished with questions to evaluate the lifestyle and dietary behavior changes retrospectively of Kurdish population prior and post‐COVID‐19 outbreak lockdown.

## 2 MATERIALS AND METHODS

### 2.1 Study design and participants

A cross‐sectional study was carried out among a Kurdish population in Erbil, Duhok, and Sulaimani cities using Eating Behavior and Lifestyle Changes in COVID19 lockdown (EBLC‐COVID19) survey and snowball sampling in three main provinces of the Kurdistan region to obtain information regarding the influence of COVID‐19 confinement. The survey was conducted from 1st to 14th of June 2020. The study was conducted using different platforms such Salahaddin University‐Erbil email and social media on any electronic devices connected with the internet following snowball sampling method. There was no exclusion area, except for noneducated individuals, some who had to help them to fill in the form.

### 2.2 Study questionnaire and instruments

The EBLC‐COVID19 questionnaire was initially developed by previous researchers. A total of 44 questions are divided into 3 sections. (Di Renzo, Gualtieri, Cinelli, et al., 2020).

**Section I**: Socio‐demographic information (6 questions) includes age, sex, residency and employment status, weight, and height.
**Section II:** Dietary behavior data (11 questions: including two parts: a) Mediterranean diet (MD) adherence using 14‐scaled items with MD screener tool ranging from 0 to14 b) Package of structured food consumption behavior: consumption of unhealthy foods; packaged sweet and pastry products, fizzy drinks, salted snacks, and sauces used for dressing on a daily basis, meals/day).
**Section III:** Lifestyle habit change (12 questions): Physical activity, sleeping behavior, smoking habit, and shopping.

### 2.3 Ethical consent

No Ethical approval was required by university only participants consent was required and taken.

### 2.4 Statistical analysis

Various statistical analyses were performed to analyze data using SPSS statistical package for social sciences v. 21.0 (IBM). Statistical significance was set at a significant level of *p* <.05. The Shapiro–Wilk and Kurtosis test was employed to assess the normal distribution of the data. None of the group data was normally distributed. The Mann–Whitney *U* test was performed to compare the data between groups with two variables. Kruskal–Wallis test was used to compare the data between groups with more than two variables. The McNemar test was employed to correlate differences between before COVID‐19 and during lockdown categorical variables, whereas chi‐square test was performed to compare categorical variables.

## 3 RESULTS

This questionnaire was launched in 1st of June 2020, and data were collected after the lockdown was finished in the following week. The data were then coded and statistically analyzed. The total number of participants was 2,137 after data validation, and any missing data were removed. The number of participants included 74.9% (1,600) from Erbil, 22.3% (476) from Sulaimani, and 2.9% (61) from Duhok (Table 1). Regarding gender, results showed that 43.4% (927) were male and 56.4% (1,210) were female. Regarding body mass index (BMI) of the participant; healthy 50.9% (1,088), underweight 4.4% (68), overweight 33.9% (724), and obese 10.9% (232). Participants <18 years old represented the majority of the participants with 66.5% (1,422). Students 31.8% (679), employed 27.7% (592), unemployed 24.2% (518), terminated job 9.1% (195), working from home 6.4% (136), and retired 0.8% (17) were represented in the study. Regarding living place, 73.4% (1569) lived in urban areas and 26.6% (568) were from rural areas. Lastly, greater proportion of participants with a lower BMI was seen in Duhok comparing to Erbili and Sulaimani participants.

**TABLE 1:** Socio‐demographic information of participants

	Total		Erbil		Sulaimani		Duhok	
	Count	(%)	Count	(%)	Count	(%)	Count	(%)
Gender^a^								
Male	927	(43.4)	701	(32.8)	182	(8.5)	44	(2.0)
Female	1,210	(56.6)	899	(42.0)	294	(13.8)	17	(0.8)
Age^a^								
<18	1,422	(66.5)	1,072	(50.2)	308	(14.4)	42	(2.0)
18–30	616	(28.8)	460	(21.5)	140	(6.6)	16	(0.7)
31–50	52	(2.4)	33	(1.5)	18	(0.8)	1	(0.0)
>50	47	(2.2)	35	(1.6)	10	(0.5)	2	(0.1)
BMI^a^								
Underweight	93	(4.4)	68	(3.2)	22	(1.0)	3	(0.1)
Normal	1,088	(50.9)	819	(38.3)	235	(11.0)	34	(1.6)
Overweight	724	(33.9)	532	(24.9)	173	(8.1)	19	(0.9)
Obese	232	10.9)	181	(8.5)	46	(2.2)	5	(0.2)
Residency^a^								
Urban	1,569	(73.4)	1,241	(39.7)	312	(9.9)	16	(0.5)
Rural	568	(26.6)	359	(11.5)	164	(5.2)	45	(1.4)
Jobs^a^ statement								
Unemployed	518	(24.2)	365	(17.1)	141	(6.6)	12	(0.5)
Retired	17	(0.8)	11	(0.5)	6	(0.3)	0	(0.0)
Student	679	(31.8)	504	(23.6)	145	(6.7)	30	(1.4)
Working at home	136	(6.4)	95	(4.4)	37	(1.7)	4	(0.1)
Employed	592	(27.7)	471	(22.0)	108	(5.0)	13	(0.6)
Job terminated	195	(9.1)	154	(17.1)	39	(6.6)	2	(0.5)

^a^ The Shapiro–Wilk and Kurtosis test was employed to assess the normal distribution of data. None of the group data was normally distributed.

The majority of the participants declared that their lifestyle had changed in some way; 12.0% (256) of participants stated that their lifestyle was improved, whereas 50.9% (1,087) reported that their lifestyle quality had deteriorated. In particular, the frequency of physical activity was decreased and community seemed to be more sedentary (McNemar value = 60.53, *p* <.001). The percentage of people who did not exercise before and during COVID‐19 outbreak were 60% and 62.9%, respectively. Similarly, the number of training and exercise sessions per week was also significantly reduced (*p* <.001). Before the COVID‐19 emergency, the most common exercise was running, gym, and football with 14%, 9.8%, and 5.9, respectively, taking part. Whereas during the pandemic confinement, more commonly practiced sports were functional training, treadmill running, and Pilates by 30%, 4.4%, and Yoga 1.1%, respectively (Table 2).

**TABLE 2:** Types and frequency of exercise before and during COVID‐19 confinement

No	Types of exercise			
	Before	%	During	%
1	No exercise	62.9	No exercise	60.0
2	Running	14.1	Functional training	30.5
3	Gym	9.6	Treadmill	4.4
4	Football	5.8	Using training tools	1.5
5	Swimming	2.4	Yoga /Pilates	1.1
6	Aerobics	1.6	Weight lifting	0.8
7	Others	1.0	Bicycle	0.2
8	Volley ball	0.3	Gymnastics	0.1
9	Walking	0.2	Walking	0.1
10	Gymnastics	0.1		
11	Yoga/Pilates	0.1		

	Frequency of training			
	Before	%	During	%
1	No training	62.9	No training	60
2	1 to 2 times	22	1 to 2 times	25.8
3	3 to 5 times	9.1	3 to 5 times	8.7
4	More than five times	6.0	More than five times	5.5

Smoking cigarettes were slightly changing but not significantly different (McNemar value = 7.93, *p* >.05).

**FIGURE 1:**
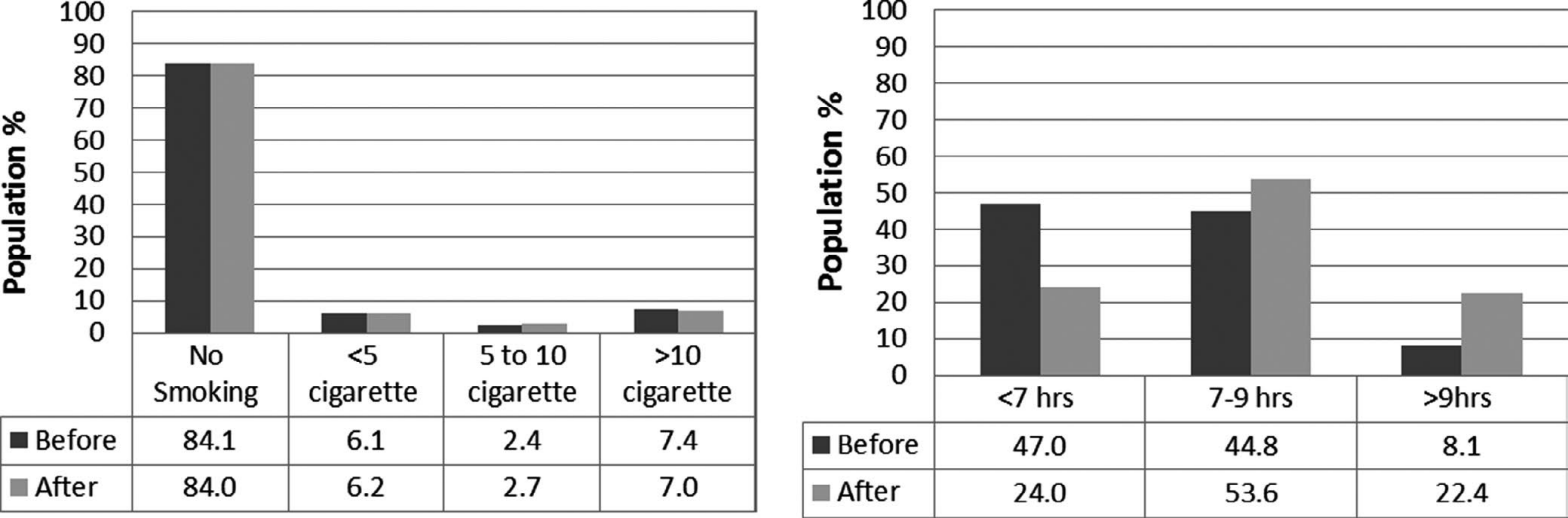
Food intake variation during COVID‐19

Regarding sleeping hours, significant differences were seen before and during COVID‐19 pandemic lockdown (McNemar value = 447.93, *p* <.001). The percentage of people reporting sleeping between 7 and 9 hr per night increased from 44.8% to 53.6% and those reporting more than 9 hr sleep increased from 8.1% to 22.4%. However, no significant differences were seen between the Erbil, Duhok, and Sulaimani (*p* =.333; Figure 1).

### 3.1 Eating habits

Regarding eating habits, the percentage of participants reporting an appetite change during COVID‐19 lockdown showed that 29.3% (624) of the participant felt appetite increased, whereas 14.3% (305) stated opposite. It was found that the percentage of participants who felt they had gained weight or gained a lot of weight was 32.4% (690) and 3.1% (67), respectively, whereas 17.9% (380) of the participants felt that they had lost weight.

The Kruskal–Wallis test revealed that the appetite change during lockdown was significantly correlated with age (*p* =.0001), gender (*p* =.001), city (*p* =.007), and BMI (*p* =.001) but not associated with participants occupation. In particular, the appetite increase was positively related age group of 18–30, males groups, normal, and overweight individuals and participants of Erbil city.

The data revealed that hunger before main meals, hunger between main meals, and hunger after dinner was stated by 52.7%, 23.6% (506), and 23.7% (507), respectively. The data also showed that 56.3% (1,204) of participants ate the same number of meals as pre COVID‐19. However, 29.2% (624) and 3.5% (74) of participated stated that they introduced or skipped one or main meal or snacks, respectively. Furthermore, the Kruskal–Wallis test also indicated that there was a significant association between perception of weight gain and age (*p* <.000), gender (*p* <.05), city (*p* < 0. 000), occupation (*p* <.05), BMI (*p* <.000), appetite (*p* <.000), and frequency of physical exercise during lockdown. In particular, participants aged 18–30 and 31–51, male participants, people from Erbil city, students and smart workers at home, overweight individuals (BMI > 25) seems to have f gained more weight during COVID‐19 lockdown.

**FIGURE 2:**
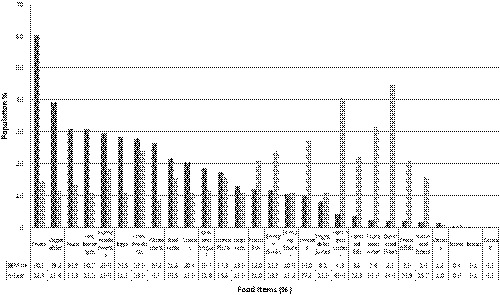
Food intake variation during COVID‐19

Food intake changes were also studied during the COVID‐19 emergency. It was found that homemade and home‐prepared foods (fruits, vegetables, homemade pizza and sweets, hot beverages, dairy products and yogurt, legumes, white meat) was increased. In contrast, there was a reduction in delivered food products (pizza and packaging sweets), processed meat, canned fish, and alcoholic intake (Figure 2).

The Kruskal–Wallis test also showed that food intake was significantly associated with age (*p* <.001), gender (*p* <.05), occupation (*p* <.05), BMI (*p* <.001), and hunger time (*p* <.05), but not related to perception of weight change, city, and appetite. Moreover, the Mann–Whitney *U* test showed that increase in unhealthy snacks (sweet beverages, packaged sweets, savory snacks, and baked products) was associated positively with age and BMI. The Mann–Whitney *U* test showed that increase unhealthy snacks was significantly associated with age groups less than 18 and 18–31 (*p* <.0001) and normal BMI (*p* <.0001). However, increase in healthy foods (legumes, vegetable, and fruits) was not significantly associated with above aforementioned variables.

Regarding the purchase place, the participants also stated that they purchased food products in supermarkets, grocery shops, and local products by 1,413 (58%), 657 (26%), and 291 (11.8%), respectively (data not shown). Despite a dramatic decrease in organic and local farming food supply chain in Kurdistan, but still 4.1% of Kurdish consumers still buy organic vegetables and fruits from local farmers due to health and safety reasons particularly during COVID‐19 pandemic.

### 3.2 MD adherence screener tool

Mediterranean diet adherence screener tool was utilized in this study as a recommended diet during COVID‐19 lockdown to discover the level of adherence of Kurdish population.

**FIGURE 3:**
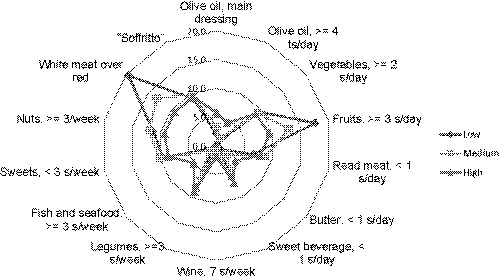
Radar plot of positive response (low, medium, and high) of MD adherence screener. The numbers are population percentage adhered to MD items in recommended servings. The values start from inner ring (0% adherence) to outer ring means higher adherence to 100%. MD, Mediterranean diet

The participants were divided into three groups based on their level of adherence to MD adherence screener into low, medium, and high adherence (Figure 3). Furthermore, the consumption e of each food item was employed using a radar chart which explains the closeness of adherence during lockdown and the ideal adherence out of 100%.

**FIGURE 4:**
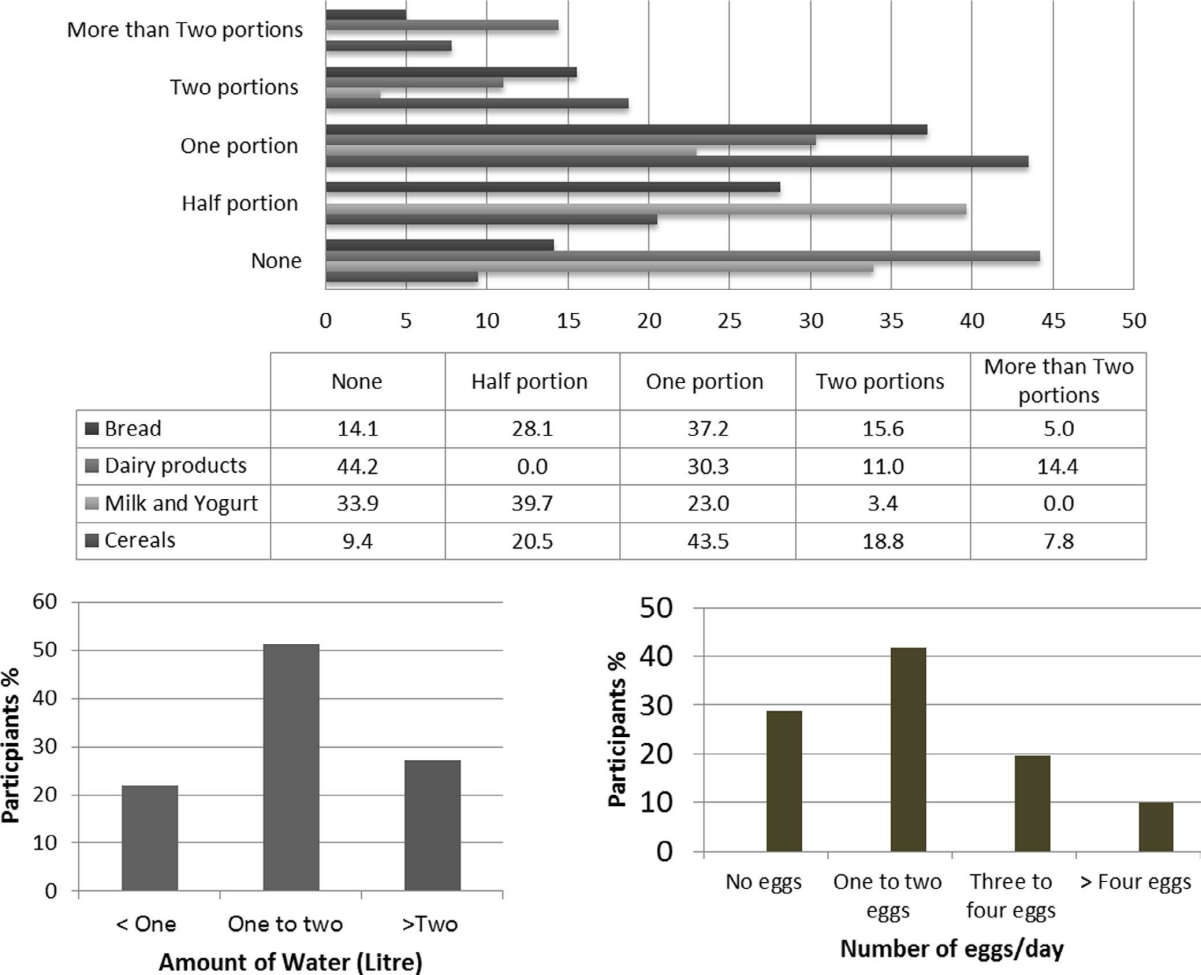
Percentage of participants consumed food and water during COVID‐19 lockdown. *Portion sizes are based on daily servings, only for dairy product weekly servings. Bread (medium portion = 80 g or 2 slices), dairy product (1 portion of = 100 g), milk, and yogurt (1 potion = 150 ml in a cup or 125 g a jar), cereals (rice, pasta, and burgul; 1 medium portion = 80 g). Eggs (number) are based on daily basis. Water (one and two) is liters based on daily basis

It can be seen that there is a large variation between consumption of the different food items. It can be seen that the highest adherence include consumption of fruits, white meat, nuts, vegetables, and legumes by 9.48%, 9.35%, 9.22%, 8.83%, and 8.83%, respectively (Figure 3). The chi‐square data analysis showed significant differences in relation to city (*p* =.04), residency (*p* =.03), and occupation (*p* =.01). Furthermore, the data showed higher positive responses among participants from Erbil city and urban areas, students. In contrast, although normal BMI and age group 18–30 seem to have greater compliance with the MD diet, but it was not significantly different. Similarly, gender differences were not significantly different. The participants’ trend in consuming serves of MD items is shown in Table 3. The majority of the participants moderately adhered to Mediterranean diet. Furthermore, intake of fruits (Duhok 83.6%, Erbil 82.6% and Sulaimani 83.0%); nuts Duhok (52.5), Erbil (68.3) and Sulaimani (72.9), vegetables Duhok (60.7%), (Erbil 55%), and (Sulaimani 54.4%) were ranked highest, respectively, among Mediterranean diet items (Table 3). In contrast, there was low adherence to olive oil consumption as a main dressing and consuming oil was seen in Erbil 305 (19.1) and 127(7.9), Duhok 8(13.1) and 7(11.5) Sulaimani 127(7.9) and 39 (8.2), respectively (Figure 4).

**TABLE 3:** Positive response to MD adherence screener during COVID‐19 lockdown

Food items	Recommended servings (day or week)	Total sample (*n* = 2,137)	Duhok (*n* = 61)	Erbil (*n* = 1,600)	Sulaimani (*n* = 476)
Vegetables	Two or more s/day	1,176 (55.0%)	37 (60.7%)	880 (55.0%)	259 (54.4%)
Fruits	Three or more /day	1,767 (82.7%)	51 (83.6%)	1,321 (82.6%)	395 (83.0%)
Olive oil	main dressing	398 (18.6%)	8 (13.1%)	305 (19.1%)	85 (17.9%)
Olive oil	Four or more ts/day	173 (8.1%)	7 (11.5%)	127 (7.9%)	39 (8.2%)
Read meat	Less than one s/day	1,026 (48.0%)	25 (41.0%)	789 (49.1%)	215 (45.2%)
Butter	Less than one s/day	268 (12.5%)	5 (8.2%)	211 (13.2%)	52 (10.9%)
Legumes	Three or more /week	979 (45.85%)	16 (26.2%)	646 (43.4%)	269 (56.5%)
Fish and seafood	Three or more s/week	183 (8.6%)	8 (13.1%)	131 (8.2%)	44 (9.2%)
Sweet beverage	Less than one /day	748 (35.0%)	20 (32.8%)	584 (36.55)	144 (30.3%)
Wine,	Seven/week	125 (5.8%)	5 (8.2%)	86 (5.4%)	34 (7.1%)
Sweets	Less than /week	1,245 (58.3%)	33 (54.1%)	954 (59.6%)	258 (54.2%)
Nuts	Three or more /week	1,471 (68.8%)	32 (52.5%)	1,092 (68.3%)	347 (72.95)
White meat over red		1,851 (86.6%)	44 (72.1%)	1,373 (85.8%)	434 (91.2%)
Pasta, rice, and burgul flavored with vegetable and spices		1,214 (56.8%)	26 (42.6)	917 (57.3%)	271 (56.9%)
Adherence to MD	High	74 (3.5%)	0 (0%)	59 (3.7%)	15 (3.2%)
	Medium	1,201 (56.2%)	30 (49.2%)	880 (55%)	291 (61.1%)
	Low	862 (40.3%)	31 (50.8%)	661 (41.3%)	170 (35.7%)

## 4 DISCUSSION

To author's best knowledge, this is the first study that has been done after the COVID‐19 lockdown in the Kurdish region, alongside the studies in other countries like Spain (Rodríguez‐Pérez et al., 2020) and Italy (Di Renzo, Gualtieri, Cinelli, et al., 2020).

During early emergence of COVID‐19 outbreak, many countries imposed protective measure to flatten positive cases including confinement and lockdown physical distance. Although these protective measures flattened the number of positive cases, but these measures as well as media bombardment news about cases and death and, losing jobs and restricting individuals freedom negatively affected psychology, life style and eating behavior of individuals and then the community. This is of particular interest when health professional recommend staying positive psychological and balanced diet important to support immune system and reduce risks of viral infection including COVID‐19 (Zhang & Liu, 2020). This is particularly important when there is no registered therapeutic or protective medication is available, and the public should be cautious and follow healthy life style and eating healthy diet.

Data showed that number of people exercise during COVID‐19 and frequency of outbreak was decreasing. Besides that, due to lockdown and joblessness, the sleeping hours are increased. These could explain that pandemics can make public more sedentary and less active particularly outdoors sport. However still, there are people who can find opportunities to do exercise at home particularly functional training (30.5%) and treadmill (4.4%). On the other hand, the number of smokers who smoked between 5 to 10 cigarettes was reduced. This could be the result of raising awareness that the smoking could worsen COVID‐19 infections in smokers (Gülsen et al., 2020).

The data also showed that nearly more than one of third of participants had perception of weight gain. This could be associated with increase in appetite and introduce one meal or snack during lockdown since nearly similar percentage of participants declared that they increased appetite and number of meals or snacks.

A previous study also found that perception of weight gain is related to change of appetite and introduce extra meals (Di Renzo, Gualtieri, Cinelli, et al., 2020).

The perception of weight gain could be correlated with the fact that people are more sedentary and less active physically and eat more frequent meals and/or snacks (Patte et al., 2016). Getting weight and/or perception of gaining weight can impart negative impact on body and psychological and well‐being of individuals on immune system. Recent studies have confirmed viral infections of COVID‐19 can be more severe among obese (Rychter et al., 2020) and stress and anxiety (Karim et al., 2020). Therefore, it is worth suggesting that besides staying healthy, psychological aspect is also important and helpful in better managing this pandemic.

Data also showed that home‐based recipe and home‐processed food consumption seem to be increasing and people spent more time preparing their own foods. Furthermore, unhealthy eating and junk foods consumption was significantly increased after quarantines in some individuals. This can be expected since the access to food supply chain of fruits and vegetables during lockdown is usually limited. In particular, in Kurdistan is a place where the majority of the fruits and vegetables are imported products and border closing with neighboring countries made it difficult to be available. This could have negative effect on consumption of immune supporting micronutrients intake and overall well‐being (Junaid et al., 2020). However, the majority of the participants moderately adhered to Mediterranean diet as a healthy and recommended diet for COVID‐19. In particular, fruits, vegetables, and in all the three cities were ranked higher intake among other items (Table 3). This number could be even higher before the quarantine since we have more access to these products. Consuming enough healthy foods can enhance nutritional status and better perform against viral infections (Silverio et al., 2020). Thus, right amount and types of diet like Mediterranean diet can provide enough amounts of immune supporting nutrients including vitamins, and minerals to ameliorate COVID‐19 virulence (Angelidi et al., 2020; Maiorino et al., 2020).

The main limitation in this research is unequal participation from the cities, age group, and job groups and more retrospective nature need to be considered. Therefore, further studies are needed to confirm these results with bigger number of participants and more equal participation of the cities and age groups. Particularly, the pandemic is ongoing and still affecting societies. Also, further measurable parameter such as real BMI blood pressure and heart rate can be important to assess.

## 5 CONCLUSIONS

This study is among the first investigations into the impact of COVID‐19 lockdown on both eating behavior and lifestyle changes and the only investigation in a Kurdish population.

The results have showed the more than half of participants are declared that COVID‐19 lockdown made their life style worse. COVID‐19 can make the community more sedentary then before the lockdown. COVID‐19 also seems eating habits has also changed negatively. Appetite and extra meals or snacks was introduced. Consequently, the perception of weight gain is prevalent among the participants. Furthermore, on the other hand the participants seem medium adhered to Mediterranean diet particularly Vegetables, fruits, and legumes are highly consumed. Therefore, a balanced die with more active lifestyle can immune supporting during pandemics like COVID‐19.

Further studies are needed to confirm these results with bigger number of participants and more balanced number of participants from the three cities, age groups, and people with different jobs. Also, further measurable parameter such as real BMI blood pressure and heart rate can be important to assess.

## CONFLICT OF INTEREST

The author declare that there is no conflict of interest.

## Data Availability

The data that support the findings of this study are available from the corresponding author by reasonable request.
